# Supplementary figures and images for: Cyclin A2 localises in the cytoplasm at the S/G2 transition to activate PLK1
Source: Life Sci Alliance. 2021 Jan 5;4(3):e202000980. doi: 10.26508/lsa.202000980 (PMC7812317; doi:10.26508/lsa.202000980)

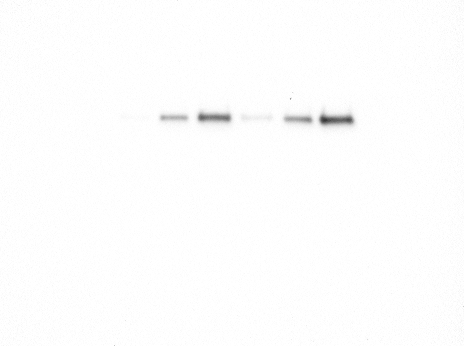

Supplement: Supplementary file 1 [file LSA-2020-00980_SdataFS3.1.tif]

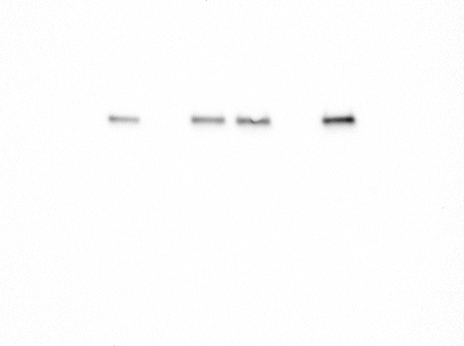

Supplement: Supplementary file 2 [file LSA-2020-00980_SdataFS3.2.tif]

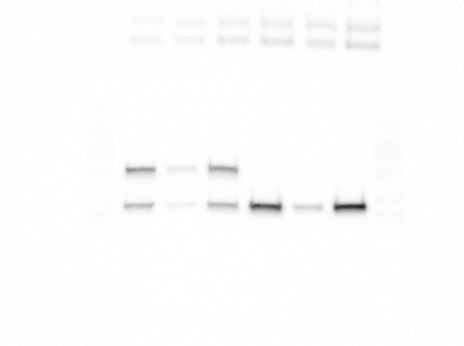

Supplement: Supplementary file 3 [file LSA-2020-00980_SdataFS3.3.tif]

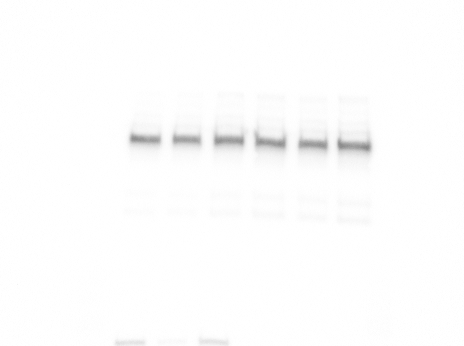

Supplement: Supplementary file 4 [file LSA-2020-00980_SdataFS3.4.tif]
