# Supplementary material for: Cyclin A2 localises in the cytoplasm at the S/G2 transition to activate PLK1
Source: Life Sci Alliance. 2021 Jan 5;4(3):e202000980. doi: 10.26508/lsa.202000980 (PMC7812317; doi:10.26508/lsa.202000980)
Supplement: Supplementary file 6 [file LSA-2020-00980_TableS1.docx]

| Name | Gene | Forward primer 5’>3’ | Reverse Primer 5’>3’ |
| --- | --- | --- | --- |
| A | MYC | GGACTCAGTCTGGGTGGAAGG | AAGGAGGAAAACGATGCCTAGA |
| B | IRF1 | GGGAGGGTTTCAGTCCTAGC | CCATCACAGCAAACCATCAA |
| C | LAMB2 | CCAGAATCCGATCATGCACC | TCCGTTTTTGCAGGTTGTGCT |
| D | α-satellite DNA | CTTTTTCATCATAGGCCTCAA | AGCTCACAGAGCTGAAACATT |
| E | Intergenic Chr1 | GCAGTTCAACCTACAAGCCAATAGAC | CACAAATTAGCGCATTGCCTGA |

Supplementary table 1.
